# Pre‐Existing and New‐Onset Atrial Fibrillation in Patients Undergoing Transcatheter Aortic Valve Implantation

**DOI:** 10.1002/ccd.70632

**Published:** 2026-04-21

**Authors:** Kimberley I. Hemelrijk, Astrid C. van Nieuwkerk, Philomene J. Vojacek, Livia Gheorge, Didier Tchétché, Fabio S. de Brito, Marco Barbanti, Ran Kornowski, Azeem Latib, Augusto D'Onofrio, Flavio Ribichini, Geoffrey Yanes Bowden, Nicolas Dumonteil, Rogerio Sarmento Leite, Samantha Sartori, Paola D'Errigo, Giuseppe Tarantini, Stefano Andreaggi, Katia Orvin, Matteo Pagnesi, Lluis Asmarats, George Dangas, Roxana Mehran, Ronak Delewi

**Affiliations:** ^1^ Department of Cardiology Amsterdam UMC Amsterdam Cardiovascular Sciences Amsterdam the Netherlands; ^2^ Hospital Puerto del mar Cadiz Spain; ^3^ Clinique Pasteur Toulouse France; ^4^ Heart Institute University of São Paulo Medical School São Paulo Brazil; ^5^ Università degli Studi di Enna “Kore” Enna Italy; ^6^ Cardiology Department Rabin Medical Center Petach Tikva Israel; ^7^ Department of Cardiology Montefiore Medical Center New York New York USA; ^8^ Department of Medicine Division of Cardiology University of Cape Town Cape Town South Africa; ^9^ Division of Cardiac Surgery University of Padova Padova Italy; ^10^ Department of Medicine Division of Cardiology University of Verona Verona Italy; ^11^ Complejo Hospitalario Universitario de Canarias Santa Cruz de Tenerife Spain; ^12^ The Zena and Michael A. Wiener Cardiovascular Institute Icahn School of Medicine at Mount Sinai New York New York USA; ^13^ National Centre for Global Health‐Istituto Superiore di Sanità Rome Italy; ^14^ Department of Medical and Surgical Specialties, Radiological Sciences and Public Health Institute of Cardiology, ASST Spedali Civili University of Brescia Brescia Italy; ^15^ Hospital de Sant Creu i Sant Pau Barcelona Spain

**Keywords:** aortic valve stenosis, atrial fibrillation, mortality, new‐onset atrial fibrillation, stroke, transcatheter aortic valve implantation

## Abstract

**Background:**

Atrial fibrillation (AF) is a frequent comorbidity in patients with severe aortic valve stenosis undergoing transcatheter aortic valve implantation (TAVI). In addition, new‐onset AF can occur after TAVI. However, data on how AF affects outcomes in patients undergoing TAVI remain conflicting.

**Aims:**

To assess clinical outcomes in patients with severe aortic valve stenosis with AF who undergo TAVI in a large real‐world global cohort.

**Methods:**

The CENTER2‐study includes 25,771 patients that underwent TAVI between 2007 and 2022. The database consists of patient‐level pooled data from 10 clinical studies. Objectives were rates of new‐onset AF ≤ 30 days, and differences in mortality and stroke according to AF status.

**Results:**

A total of 23,320 patients were included in the current analysis (56.1% female; mean age 81.5 ± 6.7 years). Pre‐existing AF was present in 28.2% (*n* = 6579) of patients. Mortality rates after TAVI were higher in patients with pre‐existing AF (19.0% vs. 14.2%, adjusted HR: 1.39, 95% CI: 1.26–1.53, *p* < 0.001). Strokes at Day 3–30 after TAVI were more frequent in patients with pre‐existing AF (1.6% vs. 1.1%, *p* = 0.004). New‐onset AF occurred in 6.2% (*n* = 681) of patients without pre‐existing AF. Mortality rates after TAVI were higher in patients with new‐onset AF (adjusted HR 1.75, 95% CI 1.24–2.49, *p* = 0.002). One‐year stroke was more frequently observed in patients with new‐onset AF after exclusion of acute periprocedural stroke (6.1% vs. 3.4%, *p* = 0.04). Major bleeding was also more frequent in patients with new‐onset AF (12.0% vs. 6.7%, *p* < 0.001).

**Conclusions:**

Patients with pre‐existing or new‐onset AF had higher mortality compared with patients without AF undergoing transfemoral TAVI. After the acute postprocedural period, 1‐year stroke rates were higher in patients with new‐onset AF.

**Trial Registration:**

ClinicalTrials.gov. Unique identifier NCT03588247.

## 1 Introduction

Transcatheter aortic valve implantation (TAVI) has become a well‐established procedure for patients with severe aortic valve stenosis. Although initially performed in inoperable and high‐risk patients, TAVI is now widely used in patients with low surgical risk [1, 2]. Atrial fibrillation (AF) is a common comorbid condition in patients who undergo TAVI [3, 4]. In addition, new‐onset AF can occur as a postprocedural complication [3, 5]. AF and aortic valve stenosis are both independently associated with increased cardiovascular mortality and morbidity, and share overlapping risk factors such as hypertension, chronic kidney disease, and advancing age [5, 6]. Pre‐existing AF as well as new‐onset AF were associated with higher rates of adverse clinical outcomes in patients who underwent cardiac surgery [7, 8]. However, data from patients with AF undergoing TAVI are conflicting and limited to small sample‐sized studies [3, 9, 10, 11]. More data about the impact of pre‐existing and new‐onset AF may improve risk stratification and patient education in this specific patient population. Therefore, we aim to assess clinical outcomes in patients with severe aortic valve stenosis with pre‐existing AF and new‐onset AF who undergo transfemoral TAVI in a real‐world global patient population.

## 2 Methods

### 2.1 Study Design and Patient Population

Patients were included in the CENTER2 study (Cerebrovascular Events in Patients Undergoing Transcatheter Aortic Valve Implantation With Balloon‐Expandable Valves vs. Self‐Expandable Valves). The initial CENTER study was a multicenter patient‐pooled analysis including 12,381 patients with severe aortic valve stenosis who underwent TAVI between 2007 and 2018 [12]. In 2022, investigators were asked to update the database with more recently treated patients which led to an addition of 13,390 patients treated and led to a total patient population of 25,771 patients in the CENTER2 [13]. The study includes patients recruited from 10 clinical studies: 2 multicenter prospective registries, 1 prospective clinical trial, 3 national registries, and 4 single‐center prospective registries (Table S1). The collaborating centers collected baseline characteristics, risk scores, echocardiographic parameters, procedural information, and long‐term follow‐up [13]. Indications for TAVI were made by the heart team of each hospital, and patients were treated with commercially available balloon‐expandable or self‐expandable valves according to contemporary guidelines. Oral anticoagulants were interrupted before the TAVI procedure, and INR was routinely checked in patients on vitamin K antagonists (VKA). Suture‐based closure devices were used. Written informed consent was provided by all patients, and the study was approved by the ethics committee of each participating center. All studies complied with the Declaration of Helsinki. The CENTER2 study is registered at clinicaltrials.gov (NCT03588247). Of the 25,771 patients included in CENTER2, 24,321 (94.4%) patients underwent transfemoral TAVI. Baseline AF status was known in 23,320 patients (95.9%) with transfemoral TAVI who were included in the current analysis.

### 2.2 Study Outcomes

The main objectives of the current study were twofold. First, we evaluated rates of 30‐day and 1‐year all‐cause mortality and stroke in patients with pre‐existing AF, compared to patients without pre‐existing AF. Second, in patients without pre‐existing AF, we assessed the incidence and clinical outcomes of new‐onset AF. All clinical outcomes were defined according to the second Valve Academic Research Consortium (VARC‐2) criteria [14].

Pre‐existing AF was defined as any type of AF recorded in a patient's medical history: paroxysmal, persistent, or permanent, detected via a 12‐lead electrocardiogram. Pre‐existing AF could be documented in a patients' medical history. Also < 24 h before TAVI, baseline electrocardiograms were made as a routine pre‐TAVI measurement.

New‐onset AF was defined according to VARC‐2 as any arrhythmia that has ECG characteristics of AF or atrial flutter and lasts sufficiently long to be recorded on a 12‐lead ECG or at least 30 s on a rhythm strip within 30 days after TAVI [14]. In addition to VARC‐2, VARC‐3 states periprocedural new‐onset AF as ≤ 30 days after the index procedure [15]. Incidence of new‐onset AF was assessed in patients without pre‐existing AF. New‐onset AF has a competing risk with mortality: patients who died periprocedurally cannot develop AF. Therefore, patients without a history of AF who were discharged alive after TAVI were included in the analysis of new‐onset AF. After TAVI, patients were continuously monitored for at least 24–72 h, according to local and contemporary guidelines. Stroke events were stratified by timing after TAVI into acute strokes occurring within 0–2 days, subacute strokes occurring between Day 3 and Day 30, and late strokes occurring beyond 30 days up to 1 year. Secondary objectives included differences in rates of adverse clinical outcomes according to AF status: 30‐day major bleeding, myocardial infarction, and permanent pacemaker implantation, according to VARC‐2 definitions [14].

### 2.3 Statistical Analysis

The distribution of continuous data was visually inspected for normal distribution. For normally distributed data, we used the independent *t*‐test, and values were reported as mean with standard deviation (SD). For non‐normally distributed data, the Mann–Whitney *U* test was used and reported as median and interquartile range. Categorical data are presented as percentages with frequencies and analyzed with the Chi‐square test or Fisher's exact test. Chi‐square test was performed to assess differences in clinical outcomes between patients with and without pre‐existing AF, and with and without new‐onset AF. One‐year mortality estimates were evaluated in a univariate and multivariate Cox regression; multivariate analyses were adjusted for baseline predictors for mortality. Hazard ratios (HR) with corresponding 95% confidence intervals (CI) were established. In patients without pre‐existing AF, independent predictors of new‐onset AF were examined using a multivariate logistic regression analysis: all baseline patient characteristics were tested as univariate predictors, and those with *p* < 0.1 were combined in a multivariate model to determine adjusted odds ratio (OR) and 95% CI. We performed sensitivity analyses to assess new‐onset AF incidence in patients with non‐transfemoral TAVI access. Additional sensitivity analyses were conducted after exclusion of patients with acute periprocedural stroke occurring within 0–1 day. All *p* values were two‐sided, and values of *p* < 0.05 were considered statistically significant. Calculations were performed using SPSS Statistics (Version 28.0 for Windows, SPSS Inc, Chicago, IL, USA).

## 3 Results

### 3.1 Baseline Characteristics in Patients With and Without Pre‐Existing AF

A total of 23,320 patients underwent transfemoral TAVI, with a mean age of 81.5 ± 6.7%, and 56.1% was female. A total of 6579 (28.2%) of these patients had pre‐existing AF. Table 1 presents baseline characteristics of patients with pre‐existing AF and patients without AF. Patients with pre‐existing AF were slightly older and more frequently male. Patients with pre‐existing AF had a higher prevalence of comorbidities: previous cerebrovascular events, peripheral vascular disease, hypertension, and renal failure.

**Table 1:** Baseline characteristics of patients with and without pre‐existing AF.

	Total (*n* = 23,320)	No pre‐existing AF (*n* = 16,741)	Pre‐existing AF (*n* = 6579)	*p* value
Demographics				
Age^b^	81.5 ± 6.7	81.3 ± 7.0	81.9 ± 6.1	< 0.001
Woman	13,079 (56.1)	9470 (56.6)	3609 (54.9)	0.02
Body mass index (kg/m^2^)^b^	27.5 ± 4.9	27.5 ± 4.9	27.4 ± 4.9	0.46
Medical history				
Cerebrovascular events	2367 (10.2)	1539 (9.3)	828 (12.7)	< 0.001
Myocardial infarction	2927 (12.9)	2155 (13.1)	772 (12.1)	0.04
Previous CABG	2048 (9.0)	1528 (9.4)	520 (8.1)	0.003
Previous PCI	4758 (20.8)	3626 (22.1)	1132 (17.7)	< 0.001
Peripheral vascular disease	2811 (12.2)	1975 (11.9)	836 (12.9)	0.03
Diabetes mellitus	7592 (32.8)	5440 (32.7)	2152 (32.9)	0.79
Hypertension	18,825 (81.0)	13,432 (80.5)	5393 (82.4)	< 0.001
Dyslipidemia	13,544 (58.5)	9858 (59.3)	3686 (56.4)	< 0.001
Coronary artery disease	8624 (37.8)	6395 (39.0)	2229 (34.7)	< 0.001
Permanent pacemaker	1193 (8.2)	1082 (10.3)	111 (2.7)	< 0.001
Renal failure (eGFR< 30)	2591 (12.4)	1811 (12.1)	780 (13.2)	0.03
eGFR (mL/min/1.73m^2^)^a^	52.6 (38.9–69.3)	53.0 (39.1–69.8)	51.0 (37.9–67.8)	< 0.001
NYHA III or IV	8228 (50.8)	5772 (49.2)	2456 (55.0)	< 0.001
Risk scores				
STS‐PROM (%)^a^	4.9 (3.1–8.5)	4.7 (3.0–8.2)	5.3 (3.3–9.2)	< 0.001
EuroSCORE I (%)^a^	13.5 (8.5–21.4)	12.8 (8.1–20.4)	15.5 (9.6–24.0)	< 0.001
EuroSCORE II (%)^a^	3.6 (2.2–6.0)	3.5 (2.1–5.6)	4.1 (2.6–6.8)	< 0.001
CHA_2_DS_2_VASc^b^	4.5 ± 1.1	4.5 ± 1.1	4.6 ± 1.0	< 0.001
Echocardiographic parameters				
Aortic valve area (cm^2^)^b^	0.67 ± 0.20	0.67 ± 0.20	0.67 ± 0.19	0.11
Max gradient (mmHg)^b^	77.8 ± 23.2	79.1 ± 23.3	74.9 ± 22.9	< 0.001
Mean gradient (mmHg)^b^	48.8 ± 15.7	49.9 ± 16.6	46.9 ± 16.0	< 0.001
LVEF (%)^b^	56.9 ± 13.1	57.2 ± 13.1	55.8 ± 13.2	< 0.001
Balloon‐expandable valve	9955 (42.7)	7014 (41.9)	2941 (44.7)	< 0.001
Antithrombotic treatment at discharge				
None	49 (0.3)	27 (0.3)	22 (0.5)	0.01
Single antiplatelet	3055 (21.6)	2641 (26.3)	414 (10.1)	< 0.001
Double antiplatelet	5842 (41.3)	5327 (53.0)	515 (12.6)	< 0.001
Oral anticoagulation	5202 (36.8)	2062 (20.5)	3140 (76.8)	< 0.001
DOAC	1340 (15.8)	539 (9.6)	801 (31.4)	0.002
VKA	1777 (21.0)	619 (10.9)	1159 (50.1)	0.002

*Note:* Values are numbers (percentages).

Abbreviations: CABG, coronary artery bypass grafting; DOAC, direct oral anticoagulant; eGFR, estimated glomerular filtration rate; EuroSCORE, European System for Cardiac Operative Risk Evaluation; LVEF, left ventricular function; NYHA, New York Heart Association; PCI, percutaneous coronary intervention; STS‐PROM, Society of Thoracic Surgeons Predicted Risk of Mortality; VKA, vitamin K antagonist.

a Median (interquartile range).

^b^ Mean ± standard deviation.

### 3.2 Clinical Outcomes in Patients With and Without Pre‐Existing AF

**Figure 1:**
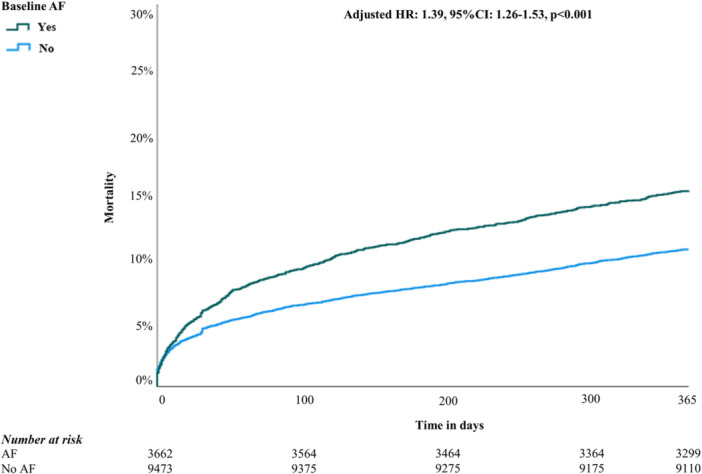
Time to mortality estimates of patients with versus without pre‐existing atrial fibrillation. Time to mortality curves for baseline with baseline atrial fibrillation (green line) versus without (blue line). Estimates were adjusted for sex, age, body mass index, peripheral vascular disease, renal failure, dyslipidemia, hypertension, and prior myocardial infarction. [Color figure can be viewed at wileyonlinelibrary.com]

Table 2 displays clinical outcomes in patients with pre‐existing AF and patients without AF. Patients with pre‐existing AF had higher rates of 30‐day mortality than patients without AF (5.9% vs. 4.5%, OR 1.33, 95% CI 1.16–1.52, *p* < 0.001). Rates of 1‐year mortality were also higher in patients with AF (19.0% vs. 14.2%, HR 1.42, 95% CI 1.30–1.55, *p* < 0.001). The adjusted HR for mortality was also higher in patients with pre‐existing AF (adjusted HR: 1.39, 95% CI 1.26–1.53, *p* < 0.001). Figure 1 presents the time to mortality curves of patients with and without pre‐existing AF. There were no differences in stroke rates at 30‐day (2.4% vs. 2.2%, OR 1.08, 95% CI 0.89–1.30, *p* = 0.47) and 1‐year follow‐up (7.2% vs. 6.9%, OR 1.05, 95% CI 0.89–1.24, *p* = 0.57) in patients with and without pre‐existing AF. When stratified by stroke timing, acute stroke was more frequent in patients without pre‐existing AF (0.8% vs. 1.1%, OR 0.73, 95% CI 0.53–0.98, *p* = 0.04). However, subacute stroke was more frequent in patients with pre‐existing AF (1.6% vs. 1.1%, OR 1.44, 95% CI 1.13–1.84, *p* = 0.004). Rates of late stroke were comparable between groups (2.0% vs. 2.1%, OR 0.96, 95% CI 0.71–1.29, *p* = 0.78). After exclusion of acute periprocedural stroke, stroke rates were similar between patients with pre‐existing AF at 30 days (0.9% vs. 0.7%, OR 1.27, 95% CI 0.94–1.74, *p* = 0.14) and at 1 year (4.2% vs. 3.8%, OR 1.11, 95% CI 0.98–1.38, *p* = 0.37). Patients with pre‐existing AF more frequently had a permanent pacemaker implanted (19.9% vs. 17.1%, OR 1.21, 95% CI 1.07–1.36, *p* = 0.002). Rates of major bleeding and myocardial infarction were similar in both groups.

**Table 2:** Clinical outcomes in patients with and without pre‐existing AF.

	Total (*n* = 23,320)	No pre‐existing AF (*n* = 16,741)	Pre‐existing AF (*n* = 6579)	Odds ratio (95% CI)	*p* value
Length of hospital stay (days)^a^	6 (4–9)	7 (5–10)	6 (4–9)	**—**	< 0.001
Thirty days					
All‐cause mortality	1009 (4.9)	670 (4.5)	339 (5.9)	1.33 (1.16–1.52)	< 0.001
Stroke	522 (2.2)	367 (2.2)	155 (2.4)	1.08 (0.89–1.30)	0.47
Permanent pacemaker	1652 (17.8)	1171 (17.1)	481 (19.9)	1.21 (1.07–1.36)	0.002
Myocardial infarction	204 (1.1)	152 (1.1)	52 (1.0)	0.90 (0.66–1.24)	0.53
Major bleeding	1514 (6.7)	1102 (6.8)	412 (6.5)	0.95 (0.84–1.07)	0.37
One year					
All‐cause mortality	2162 (15.6)	1412 (14.2)	750 (19.0)	1.42 (1.30–1.55)^a^	< 0.001
Stroke	740 (7.0)	529 (6.9)	211 (7.2)	1.05 (0.89–1.24)	0.57

*Note:* Hazard ratio was reported for one‐year all‐cause mortality. Values are number (percentage) or ^a^median (interquartile range).

### 3.3 New‐Onset AF

After transfemoral TAVI, 16,045 patients (95.8%) without a history of pre‐existing AF were discharged alive after TAVI. A total of 681 (6.2%) developed new‐onset AF post‐TAVI. Table 3 displays baseline characteristics of patients with and without new‐onset AF. Patients with new‐onset AF were slightly older and more frequently had a NYHA functional class III or IV. Incidence of new‐onset AF was more frequent in patients that had a balloon‐expandable valve implanted. New‐onset AF incidence was similar in women and men. In a multivariate analysis, independent baseline predictors of new‐onset AF were increasing age (OR 1.03 per year, 95% CI 1.01–1.06, *p* = 0.001) and NYHA functional class III or IV (OR 1.54, 95% CI 1.19–1.98, *p* < 0.001) (Table 4).

**Table 3:** Baseline characteristics of patients with and without new‐onset atrial fibrillation.

	No AF (*n* = 10,274)	New‐onset AF (*n* = 681)	*p* value
Demographics			
Age	81.4 ± 7.0	82.1 ± 6.7	0.01
Female	5868 (57.1)	393 (57.7)	0.76
Body mass index (kg/m^2^)	27.3 ± 4.9	27.3 ± 5.2	0.89
Medical history			
Cerebrovascular events	917 (9.0)	73 (10.8)	0.12
Myocardial infarction	1369 (13.6)	75 (11.1)	0.06
Previous CABG	1032 (10.3)	63 (9.3)	0.43
Previous PCI	2276 (22.5)	136 (20.0)	0.14
Peripheral artery disease	1285 (12.7)	89 (13.2)	0.71
Diabetes mellitus	471 (31.8%)	207 (30.5%)	0.48
Hypertension	8174 (79.8)	555 (81.7)	0.22
Dyslipidemia	5815 (56.9)	392 (58.0)	0.58
Coronary artery disease	4156 (41.1)	240 (35.9)	0.01
Permanent pacemaker	648 (11.0)	24 (5.6)	< 0.001
Renal failure (eGFR < 30)	1177 (12.6)	73 (15.3)	0.08
eGFR (mL/min/1.73 m^2^)	51.9 (38.2–68.0)	52.8 (37.6–68.1)	0.72
NYHA III or IV	3201 (47.9)	303 (55.0)	0.001
Risk scores			
STS‐PROM (%)	5.0 (3.2–9.5)	4.8 (3.1–‐8.6)	0.29
EuroSCORE I (%)	13.1 (8.5–21.0)	14.5 (9.1–23.2)	0.01
EuroSCORE II (%)	3.6 (2.2–5.7)	3.2 (2.1–5.3)	0.14
CHA_2_DS_2_VASc	4.5 ± 1.1	4.4 ± 1.0	0.08
Echocardiographic parameters			
Aortic valve area (cm^2^)	0.67 ± 0.20	0.69 ± 0.19	0.02
Max gradient (mmHg)	79.0 ± 23.5	82.2 ± 24.0	0.01
Mean gradient (mmHg)	50.2 ± 17.1	51.2 ± 16.4	0.15
LVEF %	57.2 ± 13.3	57.6 ± 12.2	0.51
Balloon‐expandable valve	4429 (43.1)	258 (37.9)	0.01
Antithrombotic treatment at discharge			
None	23 (0.4)	0	0.16
Single antiplatelet	1696 (26.7)	115 (21.3)	0.005
Double antiplatelet	3435 (54.1)	163 (30.1)	< 0.001
Oral anticoagulation	1190 (18.8)	263 (48.1)	< 0.001
DOAC	224 (8.2)	96 (25.0)	0.047
VKA	292 (10.6)	89 (23.1)	0.047

*Note:* Values are presented as numbers (percentages), mean (standard deviation) or median (interquartile range).

Abbreviations: CABG, coronary artery bypass grafting; DOAC, direct oral anticoagulant; eGFR, estimated glomerular filtration rate; Logistic EuroSCORE, Logistic European System for Cardiac Operative Risk Evaluation; LVEF, left ventricular function; NYHA, New York Heart Association; PCI, percutaneous coronary intervention; STS‐PROM, Society of Thoracic Surgeons Predicted Risk of Mortality; VKA, vitamin K antagonist.

**Table 4:** Predictors of new‐onset atrial fibrillation.

	Univariate model		Multivariate model	
	Odds ratio (95% CI)	*p* value	Odds ratio (95% CI)	*p* value
Age (per year)	1.02 (1.01–1.03)	0.007	1.03 (1.01–1.06)	0.001
Previous myocardial infarction	0.79 (0.62–1.01)	0.061	0.78 (0.53–1.15)	0.21
NYHA functional class III or IV	1.33 (1.12–1.58)	0.001	1.54 (1.19–1.98)	< 0.001
Renal failure (eGFR < 30)	1.26 (0.97–1.62)	0.083	1.09 (0.77–1.55)	0.63
Aortic valve area	1.63 (1.08–2.47)	0.021	1.52 (0.80–2.91)	0.20
Balloon‐expandable valve	0.81 (0.69–0.95)	0.008	1.14 (0.90–1.45)	0.31

Abbreviations: CI, confidence interval; eGFR, estimated glomerular filtration rate; NYHA, New York Heart Association.

### 3.4 Clinical Outcomes in Patients With and Without New‐Onset AF

Table 5 presents clinical outcomes in patients with new‐onset AF and patients without AF. No significant differences were seen in mortality at 30‐day (1.1% vs. 0.6%, OR 1.84, 95% CI 0.84–4.04, *p* = 0.13) and 1‐year follow‐up (11.1% vs. 8.6%, HR 1.27, 95% CI 0.94–1.73, *p* = 0.13) between patients with new‐onset AF and without AF. However, after adjustment for comorbidities, new‐onset AF was associated with higher mortality (adjusted HR 1.75, 95% CI 1.24–2.49, *p* = 0.002). Stroke incidence was not different at 30 days (2.2% vs. 2.1%, OR 1.03, 95% CI 0.61–1.75, *p* = 0.92) nor at 1 year (8.1% vs. 6.0%, OR 1.38, 95% CI 0.88–2.16, *p* = 0.16). When stratified by stroke timing, acute stroke did not differ between patients with and without new‐onset AF (1.1% vs. 0.7%, OR 0.67, 95% CI 0.27–1.63, *p* = 0.37). Similarly, subacute stroke (1.5% vs. 1.0%, OR 1.42, 95% CI 0.74–2.72, *p* = 0.29) and late stroke (2.6% vs. 2.1%, OR 1.21, 95% CI 0.56–2.63, *p* = 0.62) were comparable between groups. After exclusion of acute periprocedural stroke, 30‐day stroke was similar between patients with and without new‐onset AF (1.3% vs. 0.7%, OR 1.89, 95% CI 0.94–3.79, *p* = 0.10). However, 1‐year stroke was more frequently observed in patients with new‐onset AF compared to patients without new‐onset AF (6.1% vs. 3.4%, OR 1.81, 95% CI 1.07–3.07, *p* = 0.04). Patients with new‐onset AF more frequently had major bleeding (12.0% vs. 6.7%, OR 1.90, 95% CI 1.49–2.43, *p* < 0.001) compared to patients without new‐onset AF. They more often required permanent pacemaker implantation (25.2% vs. 15.3%, OR 1.03, 95% CI 0.61–1.75, *p* < 0.001).

**Table 5:** Clinical outcomes in patients with and without new‐onset atrial fibrillation.

	No AF (*n* = 10,274)	New‐onset AF (*n* = 681)	Odds ratio (95% CI)	*p* value
Length of hospital stay (days)^a^	7 (5–10)	8 (6–12)	—	< 0.001
At 30 days				
All‐cause mortality	57 (0.6)	7 (1.1)	1.84 (0.84–4.04)	0.13
Stroke	220 (2.1)	15 (2.2)	1.03 (0.61–1.75)	0.92
Permanent pacemaker implantation	825 (15.3)	61 (25.2)	1.86 (1.38–2.51)	< 0.001
Myocardial infarction	87 (1.0)	10 (1.7)	1.73 (0.89–3.34)	0.10
Major bleeding	673 (6.7)	81 (12.0)	1.90 (1.49–2.43)	< 0.001
At 1 year				
All‐cause mortality	590 (8.6)	45 (11.1)	1.27 (0.94–1.73)^b^	0.13
Stroke	338 (6.0)	22 (8.1)	1.38 (0.88–2.16)	0.16

*Note:* Values are numbers (percentages).

Abbreviations: AF, atrial fibrillation; CI, confidence interval.

^a^ Median (interquartile range).

^b^ Hazard ratio was reported for 1‐year all‐cause mortality.

### 3.5 Comparison Between Types of AF

**Figure 2:**
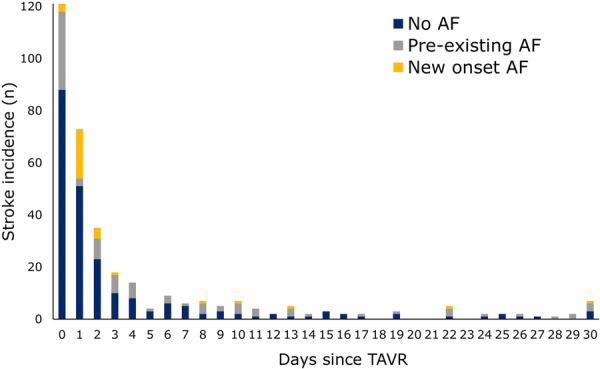
Stroke incidence in patients undergoing TAVI in patients with and without atrial fibrillation. Blue bars indicate stroke rates per day after TAVI in patients without AF; green bars in patients with pre‐existing AF, and red bars in patients with new onset AF. [Color figure can be viewed at wileyonlinelibrary.com]

Rates of 30‐day stroke were comparable between pre‐existing and new‐onset AF (2.4% vs. 2.5%, OR 0.95, 95% CI 0.58–1.56, *p* = 0.80). One‐year stroke rates did not differ significantly (7.9% vs. 11.1%, OR 0.86, 95% CI 0.56–1.32, *p* = 0.49). Figure 2 presents stroke incidence per day after TAVI according to AF status.

### 3.6 Anticoagulant Strategies

Only 76.8% (*n* = 3140) of patients with pre‐existing AF were discharged on oral anticoagulation. However, stroke rates did not differ between patients with AF who received anticoagulation at discharge and those without anticoagulation at 30 days (2.5% vs. 2.0%, *p* = 0.33) or at 1 year (6.5% vs. 6.4%, *p* = 0.98). There was no significant difference in stroke rates among patients receiving DOACs, VKAs, or no anticoagulation (1.7% vs. 1.5% vs. 2.0%, *p* = 0.28). However, at 1 year, patients treated with DOACs had a higher incidence of stroke compared with the other groups (10.4% vs. 6.2% vs. 6.7%, *p* = 0.03). Use of VKA was associated with higher bleeding rates than DOAC (8.2% vs. 4.6%, OR 1.83, 95% CI 1.35–2.49, *p* < 0.001).

Half of patients (48.1%) with new‐onset AF were discharged on anticoagulation. There was no difference in stroke rates in patients with new‐onset AF who received anticoagulation at discharge and those without anticoagulation at 30 days (1.1% vs. 1.0%, *p* = 0.96) and 1‐year (3.3% vs. 17.6%, *p* = 0.09). There were no statistically significant differences in stroke rates in patients with or without anticoagulation (1.8% vs. 2.7%, *p* = 0.50), nor in major bleeding (11.0% vs. 13.4%, *p* = 0.39).

## 4 Discussion

This global multicenter study, including > 20,000 patients undergoing transfemoral TAVI, demonstrated the following: First, pre‐existing AF was present in 28.2% of patients and was associated with a higher incidence of subacute stroke and with increased 1‐year mortality compared with patients without AF. Second, among patients without pre‐existing AF, 6.2% developed new‐onset AF, which was associated with higher rates of mortality and major bleeding at 1 year. Last, older age and higher NYHA class were independent predictors for new‐onset AF in patients undergoing transfemoral TAVI.

### 4.1 Patients With Pre‐Existing AF

Pre‐existing AF was present in 28.2% of the patients, which is in line with 15%–41% reported in previous TAVI trials [1, 2, 3, 16, 17, 18, 19]. In our study, patients with pre‐existing AF were older, had reduced left ventricular ejection fraction, and higher risk scores compared to patients without pre‐existing AF. We found increased mortality rates in patients with pre‐existing AF compared to those without AF. This increased mortality rate may be attributed to cardiac dysfunction and impaired overall functional status in patients with AF [20]. Accordingly, AF is better considered a marker of disease severity, rather than a direct cause of mortality. Higher rates of baseline comorbidities were also represented by higher predicted risk of mortality with STS‐PROM and EuroSCORE. AF is included as a risk factor in STS‐PROM, but not in EuroSCORE. Our data support the inclusion of AF as an independent factor in TAVI risk scores for mortality. Left ventricular dysfunction leads to a fivefold higher risk for AF [20]. Indeed, left ventricular ejection fraction was lower in patients with AF. Tachycardia‐induced cardiomyopathy leads to ventricular dysfunction, and loss of the atrial contraction can further impair hemodynamics [20]. In patients with severe aortic valve stenosis, chronic volume and pressure overload can facilitate the persistence of AF. After restoration of valve dysfunction and pressure overload by TAVI, attempts for rhythm control may theoretically be more successful. However, this has not been proven in a clinical study.

Early stroke after TAVI is strongly associated with procedural factors, such as debris dislodgement during the valve implantation, and this relationship appears to be independent of AF status. In contrast, late strokes tend to be more related to patient‐specific factors, including underlying AF, ventricular dysfunction, or other cardioembolic sources, indicating that cardioembolism plays a more significant role in late strokes post‐TAVI [12]. On the contrary, late strokes are generally more of a thromboembolic origin and strongly related to patient characteristics [21].

### 4.2 Patients With New‐Onset AF

A total of 6.2% of the patients developed new‐onset AF after transfemoral TAVI. This is on the lower end of the spectrum, yet in line with several previous studies [3, 11, 17]. After adjusting for relevant comorbidities, the HR for mortality was higher in patients with new‐onset AF. New‐onset AF after TAVI may occur more frequently in frail patients with hemodynamic instability or periprocedural complications. In this data set, not all of these factors were captured, and therefore, they could not be included in the multivariable adjustment. Consequently, AF may represent an important marker of frailty rather than a direct cause of mortality. Prior studies evaluating the association between new‐onset AF and stroke after TAVI have reported inconsistent results [3, 10, 17, 18]. It has been hypothesized that subacute strokes may be due to (silent) new‐onset AF [9]. However, patients are continuously monitored during admission to detect postprocedural conduction and rhythm disturbances. After exclusion of acute periprocedural stroke, patients with new‐onset AF had a higher 1 year stroke rate. Several mechanisms may explain this finding. First, new‐onset AF occurred more frequently in patients with a higher clinical risk profile, who may already be predisposed to thromboembolic events. Second, AF itself is a well‐established risk factor for thromboembolic events. The observed association may therefore reflect both the higher baseline risk of these patients and the additional embolic risk related to AF, especially given the relatively low rates of oral anticoagulation observed in patients with new‐onset AF.

In the current study, higher NYHA functional class and higher age were independent predictors of new‐onset AF. Age as a predictor of new‐onset AF is consistent with the association of higher age and increasing prevalence of AF in the general population, especially in patients > 65 years, which covers the great majority of patients undergoing TAVI [20]. Also, more severe heart failure is a well‐known risk factor for AF in general [20, 22].

### 4.3 Antithrombotic Management

Only four out of five patients with pre‐existing AF had oral anticoagulation at discharge. These low rates of anticoagulation therapy may reflect the real‐world practice and the frailty of the AF patient population undergoing TAVI. Stroke rates were similar in patients with and without anticoagulation, which may indicate that treatment decisions reflected an individualized balance between ischemic and bleeding risk.

However, major bleeding rates were higher in patients with new‐onset AF than in those without new‐onset AF. These findings are in line with previous studies and directly relate to the use of anticoagulants [10, 11]. However, only half of patients with new‐onset AF were discharged on anticoagulation consistent with previous studies [5, 16, 23]. In fact, postprocedural bleeding complications may trigger new‐onset AF, but are also a contraindication for anticoagulation.

Guidelines recommend the use of DOAC or VKA in patients with AF and bioprosthetic heart valves [24, 25], but no specific recommendations for the type of anticoagulation in patients undergoing TAVI are available. Patients with AF receiving DOACs experienced a lower incidence of major bleeding compared to those treated with VKA. Although these findings should be interpreted cautiously, they align with observational data from the STS‐TVT registry, which also indicated reduced major bleeding rates with DOAC therapy [26]. Another propensity‐matched cohort study observed similar bleeding rates between the two groups but noted a higher ischemic risk associated with DOACs, primarily driven by non‐disabling strokes [27]. However, comparing DOACs to VKAs in real‐world settings carries a risk of selection bias. In the randomized ENVISAGE‐TAVI AF trial, edoxaban was linked to increased major bleeding rates compared to VKA, with no significant difference in stroke reduction [28]. Similarly, the ATLANTIS trial, which randomized patients requiring anticoagulation to either apixaban or VKA, found no significant differences in primary or secondary outcomes, including mortality, bleeding, and stroke [29].

Additionally, our findings indicated higher 1‐year stroke rates among AF patients treated with DOACs compared to those on VKA. The randomized ATLANTIS and ENVISAGE trials did not report increased rates of late stroke among patients receiving DOACs relative to those on VKAs. Therefore, our findings may be due to selection bias [29].

### 4.4 Limitations

This large study, including > 20,000 patients undergoing transfemoral TAVI, has limitations inherent to its observational design. Not all studies had central adjudication of events. A history of AF was site‐reported and did not require continuous preprocedural monitoring, nor was the duration of AF captured. Our study cannot exclude missed cases of undetected pre‐existing AF. A new paroxysm after TAVI can be mislabelled as new‐onset AF. Unmeasured confounding, such as hemodynamic instability with an independent adverse prognostic impact, may have influenced the result of this analysis. After TAVI, patients were monitored only until discharge, which may have led to underdetection of late AF and cerebrovascular events during the remaining post‐discharge period. Continuation or initiation of oral anticoagulation was per local protocol and was not captured after hospital discharge, introducing potential selection bias. Our study did not provide information regarding subtypes of AF and rate versus rhythm control as a strategy. Only patients who developed new‐onset AF within 30 days after the procedure were included in the current analysis, and late (> 30 days) new‐onset AF was not included in the current study (Central Illustration 1).

**Central Illustration 1:**
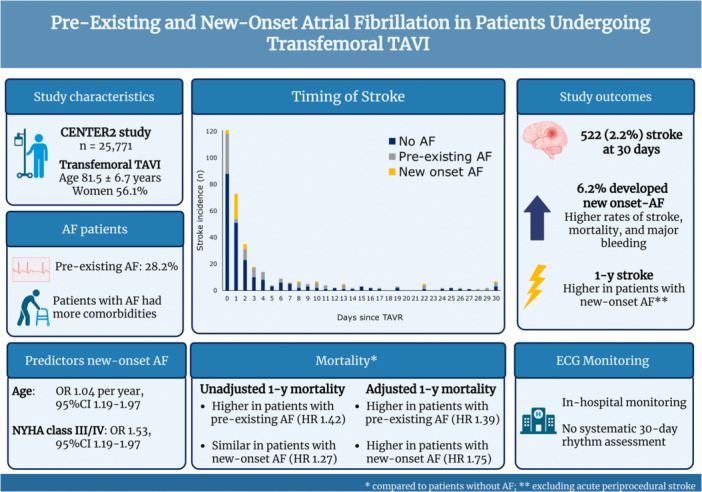
In the large real‐world CENTER2 TAVI cohort, pre‐existing atrial fibrillation (AF) was present in 28.2%, and new‐onset AF occurred in 6.2% of patients. AF was associated with higher 1‐year mortality. Patients with AF had more comorbidities, suggesting AF may represent a marker of disease severity and higher baseline risk. [Color figure can be viewed at wileyonlinelibrary.com]

## 5 Conclusions

Patients with pre‐existing or new‐onset AF had higher mortality compared with patients without AF undergoing transfemoral TAVI. After the acute periprocedural days, 1 year stroke risk was higher in patients with new‐onset AF.

## Conflicts of Interest

Dr. Fabio S. de Brito Jr is a proctor for Edwards Lifesciences and Medtronic. Dr. Marco Barbanti is a consultant for Edwards Lifesciences and received speaker honoraria from Medtronic and Biotronik. Dr. Azeem Latib is a consultant for Medtronic and received honoraria from Abbott Vascular. Dr. Matteo Pagnesi has received personal fees from Abbott Vascular. Dr. Roxana Mehran reports institutional research payments from: Abbott, Alleviant Medical, Chiesi, Concept Medical, Cordis, CPC Clinical Research, Daiichi Sankyo, Duke, Faraday Pharmaceuticals, Idorsia Pharmaceuticals, Janssen, MedAlliance, Medtronic, NewAmsterdam Pharma, Novartis, Novo Nordisk Inc., Population Health Research Institute (PHRI), Protembis GmbH, Radcliffe, RM Global Bioaccess Fund Management, Sanofi US Services Inc.; Equity < 1% in: Stel, ControlRad (spouse); Honorarium: JAMA‐JAMA Cardiology (Associate Editor), ACC (Vice President, BOT Member, SC Member CTR Program). Prof. Dr. Ronak Delewi received educational grants from Abiomed, Amgen, Meril Life, Medtronic, Sanofi, Boston Scientific and Edwards Lifesciences. The other authors declare no conflicts of interest.

## Supporting information

Supporting File

## Data Availability

The data that support the findings of this study are available from the corresponding author upon reasonable request.
