# Supplementary material for: Pre‐Existing and New‐Onset Atrial Fibrillation in Patients Undergoing Transcatheter Aortic Valve Implantation
Source: Catheter Cardiovasc Interv. 2026 Apr 21;108(1):114–23. doi: 10.1002/ccd.70632 (PMC13331581; doi:10.1002/ccd.70632)
Supplement: Supplementary file 1 — Supporting File [file CCD-108-114-s001.docx]

**Supplementary table S1. Overview of original studies included in CENTER2 with number of patients**

| Study Name | PubMed ID | Country | Inclusion period | N Included | Pre-existing AF prevalence (%) |
| --- | --- | --- | --- | --- | --- |
| Brazilian TAVI registry | 27496637 | Brazil | 2008-2015 | 768 | 14.3 |
| FRANCE-2 | 25240554 | France | 2010-2011 | 2347 | 27.6 |
| Milano | 27184169 | Italy | 2008-2015 | 515 | 32.6 |
| Verona | 27621826 | Italy | 2012-2022 | 916 | 37.6 |
| Rabin | 27726854 | Israel | 2008-2017 | 544 | 28.1 |
| Padova | 26603025 | Italy | 2007-2017 | 447 | 33.3 |
| Spanish TAVI registry | 24774108 | Spain | 2007-2022 | 4441 | 28.3 |
| BRAVO-3 | 26477635 | USA | 2012-2015 | 732 | 37.4 |
| WIN-TAVI | 27491609 | USA | 2013-2015 | 785 | 20.4 |
| OBSERVANT | 26271063 | Italy | 2008-2015 | 577 | 22.9 |
